# Placental Transfusion for Asphyxiated Infants

**DOI:** 10.3389/fped.2019.00473

**Published:** 2019-11-20

**Authors:** Anup C. Katheria, Wade D. Rich, Sunita Bava, Satyan Lakshminrusimha

**Affiliations:** ^1^Sharp Mary Birch Hospital for Women & Newborns, San Diego, CA, United States; ^2^Independent Researcher, San Diego, CA, United States; ^3^Department of Pediatrics, University of California, Davis, Davis, CA, United States

**Keywords:** placental transfusion, cord milking, delayed cord clamping, newborn, asphyxia

## Abstract

The current recommendation for umbilical cord management of non-vigorous infants (limp, pale, and not breathing) who need resuscitation at birth is to immediately clamp the umbilical cord. This recommendation is due in part to insufficient evidence for delayed cord clamping (DCC) or umbilical cord milking (UCM). These methods may provide a neuroprotective mechanism that also facilitates cardiovascular transition for non-vigorous infants at birth.

## Background

An estimated one million newborns worldwide suffer from perinatal asphyxia which lead them to being at risk for developing hypoxic-ischemic encephalopathy (HIE) due to inadequate blood flow and oxygen delivery to the neonatal brain and other vital organs such as the heart and kidneys. The incidence of HIE is 1–3/1,000 term births in high-income countries but is 15–20 times greater in low to middle-income countries. The majority of infants with severe HIE and 30–50% infants with moderate HIE either die or develop significant disabilities. In addition, subtle cognitive deficits and alterations in daily life functioning are seen even in infants with mild HIE (1).

The need for resuscitation has been identified as marker for increased risk for HIE (2–5). Improvements in delivery room management could significantly affect long-term outcomes. Helping Babies Breathe, a resuscitation algorithm developed by the American Academy of Pediatrics, has reduced mortality by training providers to provide early resuscitation with ventilation but surviving infants are still at risk for neurodevelopmental impairment. Immediate clamping and resuscitation has been the standard of care, but this approach may be detrimental by limiting placental transfusion. Early resuscitation with positive pressure ventilation (PPV) may have benefits, but receiving additional blood through delayed cord clamping (DCC) may potentially increase intravascular volume and improve perfusion.

**Figure 1:**
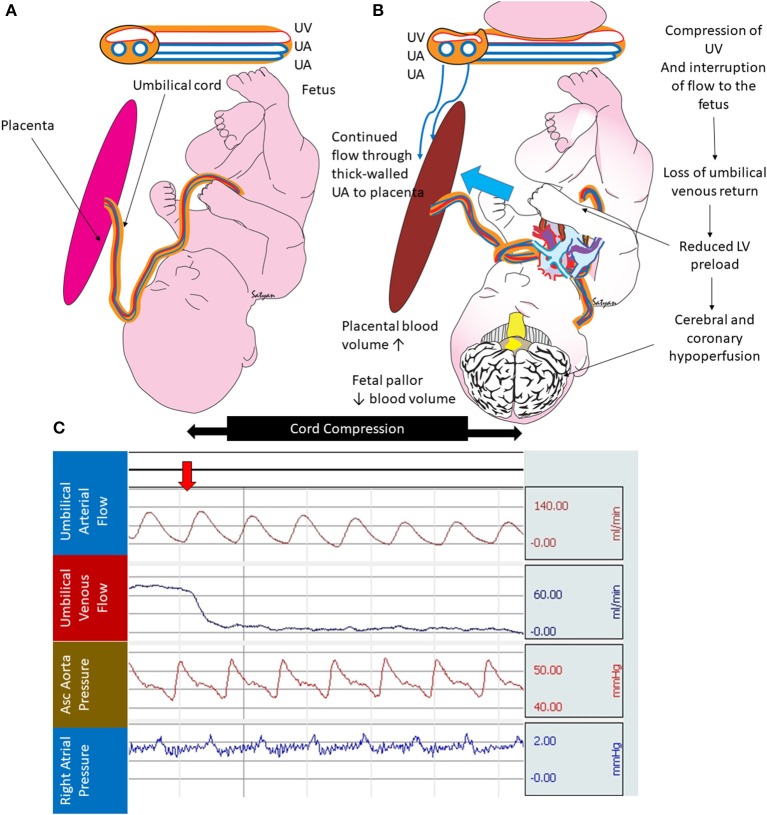
Cord compression and hypovolemia: **(A)** normal fetus with thin walled umbilical vein (UV) transferring blood from the placenta to the fetus and two thick walled umbilical arteries (UA) bringing blood from the fetus to the placenta. **(B)** Cord compression initially occludes the thin-walled UV limiting umbilical venous flow to the fetus. The thick-walled UAs continue to maintain blood flow from the fetus to the placenta (blue arrow). This process leads to increased placental blood volume and reduced fetal blood volume. **(C)** Graph from a full-term fetus showing umbilical arterial flow (persists during cord compression), umbilical venous flow (abolished after cord compression), systemic blood pressure, and right atrial pressure. Selective loss of umbilical venous flow causes hypovolemia. Copyright Satyan Lakshminrusimha, MD.

Following normal vaginal delivery, the newly born infant cries as the uterus contracts around the placenta. The combined effect of lung ventilation and uterine contraction promotes placental transfusion. Yao et al. demonstrated that blood flow continues until about 45 s after birth in the umbilical arteries while the umbilical vein remains patent until about 3 min of birth in healthy term infants (6). This results in a net transfer of blood volume from the placenta to the fetus during birth (7). However, infants at risk for asphyxia may be at risk for not receiving this transfusion at birth. Fetal blood volume loss to the placenta may occur when delivery is associated with shoulder dystocia or a tight nuchal cord (7, 8). In the presence of cord prolapse or nuchal cord, the infant as it traverses the tight birth canal, may compress the umbilical cord. In the umbilical cord, the muscular-walled, high-pressure arteries allow blood from the fetus to the placenta, while return flow from the placenta to the fetus in the thin-walled vein may be diminished or even occluded during cord compression (Figure 1). If the nuchal cord is extremely tight there may be complete occlusion of both umbilical arteries and vein in the cord.

**Figure 2:**
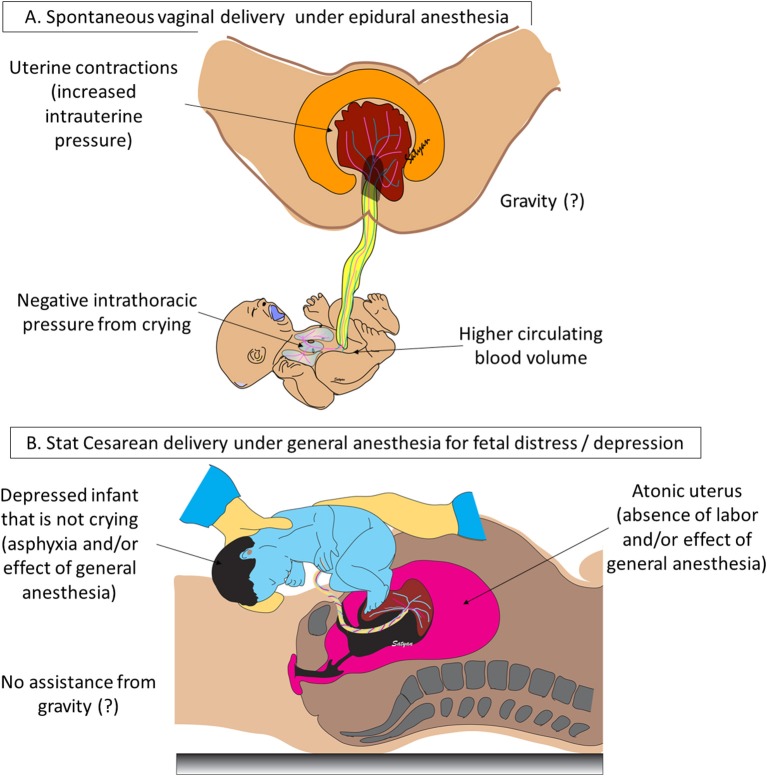
Mode of delivery, anesthesia, and neonatal vigor influence placental transfusion: **(A)** following a spontaneous vaginal delivery without general anesthesia, uterine contractions generate high intrauterine pressure (~100 mm Hg). A baby held below the introitus may benefit from gravity to enhance placental transfusion although gravity is not an absolute requirement for transfer of blood to the newly born infant. Negative intrathoracic pressure induced by active crying in a vigorous neonate can assist placental transfusion. **(B)** Following stat cesarean section under general anesthesia for fetal distress or asphyxia, uterus is atonic, infant is depressed and may not be active and baby is held at the level of the abdomen. These factors can potentially reduce the volume of placental transfusion. Copyright Satyan Lakshminrusimha, MD.

Newborns in distress are more likely to be delivered by cesarean delivery (Figure 2). These infants are also less likely to get an adequate transfer of blood even with DCC. The intact contracting uterus is the largest driving force of a placental transfusion (up to 100 mm Hg). Aladangady et al. reported lower circulating red cell volume with DCC in neonates delivered by cesarean section compared to vaginal delivery (9). They also found that blood volume increased as duration of DCC was prolonged up to 60 s in infants with vaginal delivery, but not following cesarean delivery. Strauss et al. found cesarean section delivered newborns who received DCC for 60 s had decreased red cell volume compared to vaginal delivered infants (10). McDonald et al. failed to show any difference in hemoglobin levels with a 30-s DCC among infants delivered by cesarean delivery (11).

The current recommendation for umbilical cord management of infants who are depressed and need resuscitation at birth is to immediately clamp the umbilical cord. This recommendation is due in part to insufficient evidence to support DCC or umbilical cord milking (UCM) in the presence of perinatal distress (12). However, these placental transfusion methods may facilitate cardiovascular transition and be neuroprotective in non-vigorous infants at birth. This additional blood provides an increased cardiac preload before the placenta is removed from the circulation and increases blood volume, which will stabilize cardiac output and pulmonary and cerebral circulation, potentially improving further ischemia in an already compromised infant (13).

Compared to early cord clamping (ECC), both UCM and DCC have demonstrated improvements in systemic and brain perfusion, suggesting neuroprotective benefits (14, 15). DCC and/or UCM have been shown to improve heart rate, blood pressure, urine output and cerebral oxygenation, increase early hemoglobin levels, and prevent anemia in term and preterm infants without adverse effects or harm noted in any of the studies (15–24). The need for further research has been identified by the American Congress of Obstetricians & Gynecologists, which states, “infants requiring resuscitation may benefit considerably from placental transfusion, but their need for immediate attention raises questions about whether they should undergo immediate or delayed umbilical cord clamping and whether UCM may offer a unique benefit” (25).

## Hypovolemia During Asphyxia

**Figure 3:**
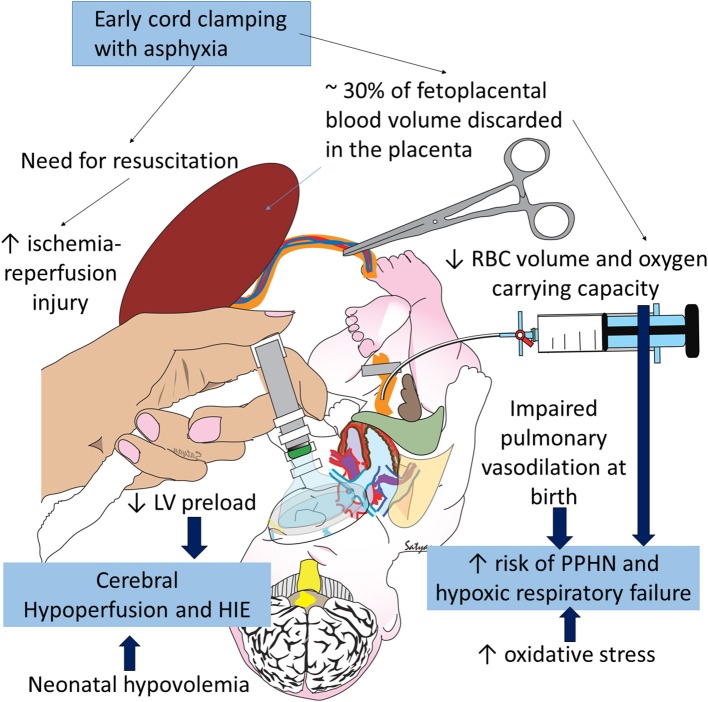
Negative consequences of early cord clamping in asphyxia: asphyxia increases the need for resuscitation and is associated with hypoxic-ischemic encephalopathy (HIE) and persistent pulmonary hypertension of the newborn (PPHN). Early cord clamping reduces blood and RBC volume in the neonate and increases fetal blood left in the placenta. Hypovolemia and hypoxia contribute to cerebral hypoperfusion and HIE and exacerbate pulmonary hypoperfusion and PPHN. Studies have shown increased oxidative stress with early cord clamping compared to delayed cord clamping and umbilical cord milking. Oxidative stress contributes to HIE and PPHN. Copyright Satyan Lakshminrusimha, MD.

When the cord is cut rapidly, the infant has no access to approximately 30 mL/kg of blood—about 30 percent of the fetal-placental blood volume in a term neonate (26)—resulting in essential hypovolemia when the lungs are first aerated after ECC (Figure 3). Placental transfusion to the infant increases blood flow to the circulatory beds while the infant's various organs (lung, liver, kidney, etc.) assume the many functions maintained by the placenta during fetal life. Losing this additional blood volume due to ECC could increase inflammatory processes and ischemia (27). In older physiologic studies comparing UCM or DCC with ECC, ECC resulted in less favorable outcomes in non-asphyxiated infants including: hypovolemia, lower blood pressures, increased vascular resistance, decreased red cell volume, less flow to brain, intestines and kidneys, lower urine output, increased sodium excretion, and lower red cell volume, hematocrit, and hemoglobin levels (28–33). These findings need confirmation in clinical trials that include asphyxiated infants.

Using the Iodine serum albumin method, Yao et al. preformed an elegant blood dilation experiment in 111 full term deliveries (26). The infants were divided into groups based on the time the umbilical cord was clamped. Combining the dilution method with placental residual blood volume (draining the placenta while it was still *in utero* and after delivery), they found that the proportion of the total fetoplacental blood volume that is in the infant/placenta was 67/33 percent at birth, 80/20 at 1 min at 87/13 at the end of the placental transfusion (about 3 min).

Lindercamp et al. looked at blood volume in 194 newborn infants (26–41 weeks) that received ECC (clamping before 15 s following vaginal birth and 5 s after Cesarean section delivery) (34). There were no differences in blood volume based on gestational age or mode of delivery (Table 1). Vaginal delivered infants with a 1 min Apgar score of ≤ 5 had a lower blood and RBC volume compared to infants with a 1 min Apgar score of >5. Infants with *intrauterine* asphyxia had much higher blood volumes (90 vs. 78 ml/kg). We speculate that infants with intrauterine asphyxia may receive a marked placental transfusion *in utero*. This may be due to the loss of systemic vasomotor response resulting in lower fetal blood pressure compared to the placenta, gasping respirations, or erythropoiesis due to chronic compromise. In sharp contrast, *intrapartum* asphyxia with a tight nuchal cord was associated with lower blood volume (67 ml/kg vs. 78 ml/kg). These results suggest that infants with intra-partum asphyxia and/or tight nuchal cords have hypovolemia (Figure 1) and may lose some blood back to the placenta. We conclude that hypovolemia is common with intrapartum asphyxia and placental transfusion may improve or restore cardiac output and systemic circulation.

**Table 1:** Changes in neonatal blood volume and RBC volume based on mode of delivery and complications.

**Mode of delivery**	**Event**	**Apgar score**	**Blood volume (ml/kg)**	**RBC volume (ml/kg)**
Vaginal delivery (15 s clamp, *n* = 141)	No complications (*n* = 96)	Apgar > 5	77.9 ± 6.2	37.5 ± 5.1
		Apgar ≤ 5	70 ± 4.4*	29.6 ± 2.9*
C-section (5 s clamp, *n* = 53)	No complications (*n* = 25)	No effect of Apgar	71.3 ± 4.8*	31.2 ± 3.6*
Intrauterine asphyxia (*n* = 56)			90.4 ± 7.0^†^	46.9 ± 6.3^†^
Intrapartum asphyxia Tight nuchal cord (*n* = 17)			67.5 ± 5.7*	27.4 ± 2.7*

Significantly lower* or higher^†^ than vaginal delivery with Apgar > 5; data from Linderkamp et al. (35).

## Breathing and Placental Transfusion (Figure 4)

A large observational study reported that newborns were more likely to be admitted to the NICU or die if their cord was clamped before they started to breathe (36). Recent data from animal studies suggests that clamping the cord before the onset of breathing leads to decreases in heart rate, right ventricular output, and pulmonary blood flow, while causing a transient spike in carotid artery blood flow (37). ECC leads to an increase in afterload and a decrease in preload, which in turn causes a significant reduction in cardiac output. Compared to UCM, preterm infants receiving ECC at birth had lower heart rates and oxygen saturation and required more oxygen and ventilation within the first 5 min of life (20). These findings highlight impaired transition from ECC in the presence of lung expansion, which likely contributed to the downstream morbidities such as increased early hypotension, number of days on oxygen, and chronic lung disease seen in the ECC group compared to UCM (38).

**Figure 4:**
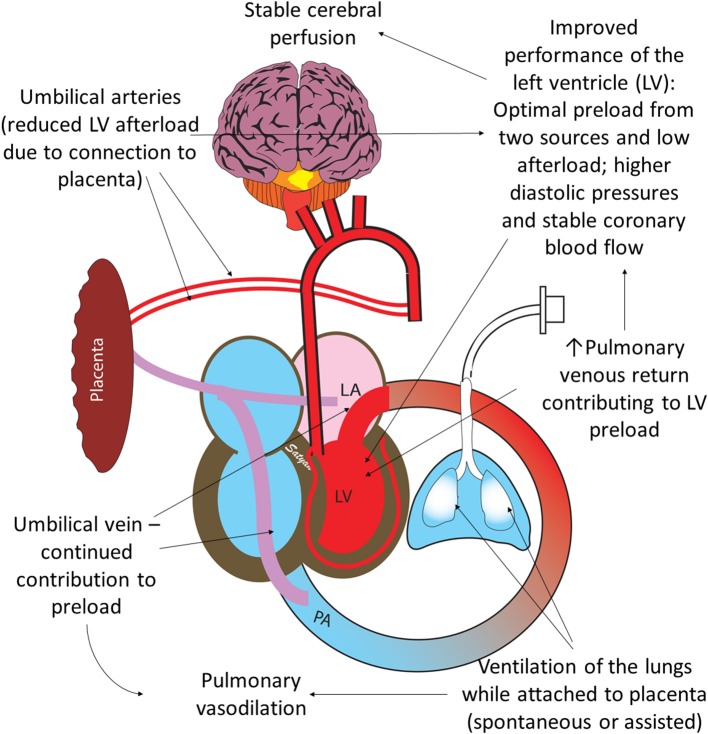
Benefits of ventilation of the lungs with an intact umbilical cord: optimal performance of the left ventricle (LV) is important for hemodynamic transition at birth. Umbilical venous return from the placenta and pulmonary venous return both contribute to LV preload. Ventilation of the lung leads to pulmonary vasodilation and increases pulmonary venous return. Umbilical arteries perfusing the placenta maintain low LV afterload. High diastolic pressure due to higher RBC volume following placental transfusion leads to better coronary perfusion. Copyright Satyan Lakshminrusimha, MD.

Optimal functioning of the left ventricle is key to normal transition at birth. Establishing ventilation of the lungs when placental circulation is still intact offers several advantages. The left ventricular afterload is low due to the presence of low-resistance umbilical circulation (Figure 4). The left ventricular preload is optimized due to dual sources of blood: umbilical venous return and pulmonary venous return (37). In addition, Davidson et al. have made some preliminary observations that diastolic pressures (an important determinant of coronary perfusion) are higher in asphyxiated lambs resuscitated with an intact cord (39).

## Asphyxia, Oxidative Stress, and PPHN

**Figure 5:**
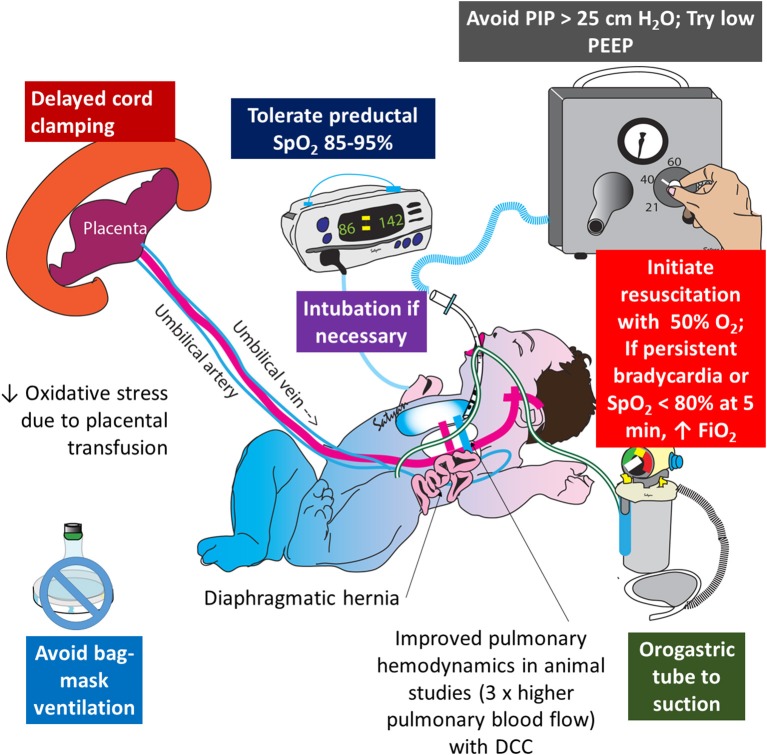
Role of delayed cord clamping in congenital diaphragmatic hernia (CDH): oxidative stress plays an important role in the pathogenesis of pulmonary hypertension and injury to hypoplastic lungs in CDH. Delayed cord clamping, limiting barotrauma (with low ventilator pressures), restricting FiO_2_ to target preductal SpO_2_ in the mid-80s to low 90s are important strategies to limit oxidative stress in CDH. Copyright Satyan Lakshminrusimha, MD.

Infants exposed to perinatal distress and birth asphyxia are at high risk of developing HIE and persistent pulmonary hypertension of the newborn (PPHN) (40). Oxidative and nitrosative stress play an important role in pathogenesis of PPHN and HIE (41). Intrapartum placental transfusion increases oxygen carrying capacity of blood and some preliminary data suggest that PPHN is associated with lower hemoglobin levels (42). A study comparing RBC catalase activity, superoxide dismutase (SOD), and total antioxidant status between ECC and DCC suggested that delayed clamping increases antioxidant capacity and reduces inflammatory effects during delivery and exerts beneficial effects on the neonate (43). DCC is also shown to increase plasma thiol levels and decrease disulfide levels in umbilical arterial blood suggesting reduced oxidative stress (44). Total antioxidant activity, RBC catalase cytosol, SOD cytosol and glutathione perioxidase cytosol are all higher with DCC along with reduced plasma hydroperioxide levels (45). DCC is being investigated in a pilot trial for infants born with congenital diaphragmatic hernia (CDH), a common cause of intractable PPHN (NCT03314233). Studies in lambs suggest that physiological/DCC reduces pulmonary vascular resistance (PVR) and significantly increases pulmonary blood flow in CDH (46). A strategy combining ventilation with lower levels of inspired oxygen (47, 48) with an intact cord might be an effective approach during the delivery room resuscitation of CDH (Figure 5).

## Animal Data to Date

The appropriateness of animal models for placental transfusion is not clear. Most of the current research involves ovine models. All of the animal models are also delivered only by Cesarean Section. Many ovine models do not use antenatal steroids which do alter the cardiopulmonary mechanics at birth following preterm delivery. The placenta in lambs is cotyledonary and the umbilical cord is relatively short. Majority of these studies are performed with an atonic uterus with a cesarean section. However, these studies provide valuable physiological data on cardiopulmonary interactions during ventilation with an intact cord.

Term lambs with asphyxia, bradycardia, and hypotension resuscitated with an intact cord demonstrated more stable cerebral perfusion and reduced cerebrovascular injury as indicated by reduced expression of blood-brain barrier protein leakage in the subcortical white matter and gray matter (49). The authors have advocated for physiologic based cord clamping, which suggests to clamp the cord when the infant has maintained stable respirations. Whether asphyxiated infants will be able to achieve stable respirations is unclear and needs to be tested prospectively in a randomized controlled trial.

The effect of PPV on placental transfusion has been evaluated in lambs. Creasy et al. did not observe an increase in neonatal blood volume following PPV with an intact cord in lambs (50). Bhatt et al. reported stable hemodynamic transition when PPV was initiated prior to cord clamping in lambs (37).

The effect of spontaneous respiration on placental transfusion is unclear. It was traditionally thought that the negative intrathoracic pressure generated by spontaneous respirations, in addition to gravity and uterine contractions, contributed to the pressure gradient from the placenta to the neonatal circulation (6, 51, 52). More recent studies in preterm lambs suggest that diaphragmatic contractions during spontaneous inspiration may compress the inferior vena cava and inhibit umbilical venous flow (53).

**Figure 6:**
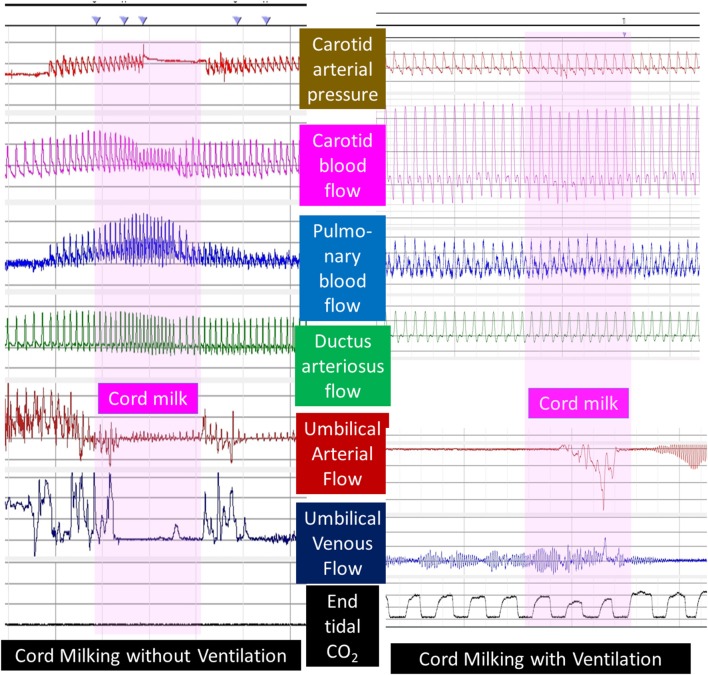
Umbilical cord milking and carotid and pulmonary hemodynamics in preterm lambs: a graph showing carotid arterial pressure, left carotid blood flow, left pulmonary arterial flow, ductus arteriosus flow, umbilical arterial, and venous flow and end-tidal CO_2_ in preterm lambs undergoing umbilical cord milking (pink vertical bar) without or with simultaneous ventilation of the lungs. Units are shown to the left and right of the figure. Interestingly, umbilical arteries went into spasm after initiation of PPV and did not demonstrate pulsatile flow. Courtesy/Copyright Praveen Chandrasekharan MD (with permission).

UCM with or without placental refill has not been shown to be beneficial in preterm lambs. Blank et al. observed significant hemodynamic disturbance with UCM in preterm lambs (54). Preliminary data by Chandrasekharan et al. suggest that fluctuations in carotid and pulmonary blood flow are common in preterm lambs without respirations receiving UCM (55). Interestingly, PPV during milking reduced fluctuations in carotid and pulmonary flow (Figure 6). Further evaluation of hemodynamic effects of milking in preterm animal models is warranted.

## Clinical Trials to Date

### Resuscitation With an Intact Cord

There are several large ongoing trials evaluating resuscitation with an intact cord to allow for a placental transfusion in a potentially asphyxiated population. Other trials that have included resuscitation on the cord but did not specifically target non-breathing or depressed infants are not discussed here.

#### Term Infants

Andersson et al. have completed a large RCT in non-breathing term infants comparing DCC with resuscitation (up to 180 s) to ECC of <60 s. They demonstrated improved Apgar scores and SpO_2_ 10 min after birth (56). Blank et al. have an ongoing trial (Blank et al., ongoing trial, Baby DUCC, Australian New Zealand trial registry Identifier: AZTN1261800621213) randomizing non-breathing infants to immediate cord clamping (<10 s) or DCC with resuscitation until they have achieved 1 min of gas exchange via a colorimetric carbon dioxide detector in the first 5 min of life.

#### Preterm Infants

Nevill et al. have an ongoing trial to randomize preterm infants not breathing well by 15 s of life and randomizing them to receive CPAP and or positive pressure ventilation or stimulation alone during DCC. Both arms will have cord clamping by 60 s of life (Nevill et al. ongoing trial, ABC trial, Australian New Zealand trial registry Identifier: AZTN12615001026516).

#### Feasibility Issues With Resuscitation With an Intact Cord

Our group has experience with conducting two trials of resuscitation with an intact cord in a term and preterm population (57, 58). For the preterm infant, most of the challenges were overcome with having adequate time to prepare for the delivery and setup the equipment. The majority of preterm infants are born by Cesarean Section; and keeping specialized cord clamping resuscitation trolley near the operating room made this feasible. There were issues regarding adequate spacing for personnel to help with the resuscitation (i.e., changing pressures during PPV), and the lack of sterile EKG leads and pulse oximetry probes to provide monitoring. For a 60 s delay in cord clamping this could be appropriate but the ongoing studies will need to include additional monitoring and personnel. The changes are necessary in order to be in line with current resuscitation guidelines which mandate adequate monitoring in infants that need resuscitation.

The most challenging population to perform resuscitation on the cord is the term asphyxiated infant. These infants do not present with enough warning since the majority of at risk term deliveries do not need resuscitation. In our pilot trial of resuscitation with an intact cord in term infants and found less than a quarter required any resuscitation (57). Our survey found resuscitation with an intact cord was challenging (59). Spacing in front of the mother during delivery with an obstetrical provider and the placement of monitoring devices was cumbersome. Further work on equipment and personnel optimization and determination whether they need to be placed in every Labor and Delivery room must occur before this method can be standard practice. The cost of these beds may be prohibitive in many countries. A low-tech option is being developed and tested in Uganda (https://www.thebabysaver.org/about-the-babysaver/) and may be an alternative.

### Cord Milking

An alternative to DCC with resuscitation is UCM. There are two methods to perform UCM. Intact cord milking (I-UCM) is performed by grasping the unclamped umbilical cord the blood is pushed (“stripped”) toward the infant two to four times before it is clamped. Cut cord milking (C-UCM) involves clamping and cutting a long segment of the umbilical cord immediately after birth and passing the baby and the long cord to the pediatrics provider. Both methods can be performed in about 20 s allowing resuscitation to take place quickly. Intact cord milking has been shown to increase the incidence of severe IVH in extremely preterm infants (<28 weeks) in a recently presented abstract (Katheria et al., ongoing trial, Premature Infants Receiving Milking or Delayed Cord Clamping: PREMOD2. ClinicalTrials.gov Identifier: NCT03019367). While there are several trials of both I-UCM and C-UCM, only studies evaluating depressed newborns will be discussed here.

#### Preterm Infants

To date, there is one small trial evaluating UCM in depressed infants. Ram Mohan et al. randomized 60 preterm infants that required resuscitation to either C-UCM or ECC. There were no differences in clinical outcomes but C-UCM infant showed higher hemoglobin and ferritin levels at 6 weeks of life (60).

#### Term Infants

There is only one trial that has evaluated UCM in depressed term infants. Girish et al. quasi-randomized (alternating months) 101 infants to either I-UCM or ECC in depressed newborns unable to receive DCC (61). There were no clinical differences in outcomes. There is one ongoing large multicenter trial (Katheria et al., ongoing trial, Milking in Non-Vigorous Infants, ClinicalTrials.org Identifier: NCT03631940) to evaluate the short and long term benefits of I-UCM in non-vigorous near term and term newborns compared to ECC.

## Conclusions

While delivery of a depressed newborn presents the challenges of a high-pressure environment, ensuring an adequate placental transfusion for these infants may be the first and most important step in ensuring the best possible outcome. Several aspects of pathophysiology of HIE and PPHN following birth asphyxia could potentially be mitigated by avoidance of ECC. The benefit of placental transfusion in such high-risk patients requires further study.

## Author Contributions

AK developed and wrote the first version of the manuscript. SB and WR revised and edited the manuscript. SL contributed animal data, new clinical studies, drew figures, and edited the manuscript.

### Conflict of Interest

The authors declare that the research was conducted in the absence of any commercial or financial relationships that could be construed as a potential conflict of interest.
